# Revision of a Hypertrophic Tracheostomy Scar

**Published:** 2016-12-22

**Authors:** Nikki Castel, Laurel Karian, Ramazi Datiashvili

**Affiliations:** Division of Plastic Surgery, Department of Surgery, Rutgers/New Jersey Medical School, Newark

**Keywords:** tracheostomy, scar, wound healing, neck muscles, contracture

## DESCRIPTION

A 43-year-old man presented with a depressed anterior neck scar from prolonged tracheostomy after crush injury. The scar caused pain and limited the range of motion of the neck. The patient underwent scar revision using a combination of strap muscle coverage and z-plasty closure.

## QUESTIONS

1. **Describe the pathogenesis of tracheostomy scars.**
2. **What is tracheal tug?**
3. **What are surgical options for hypertrophic scars?**
4. **What are surgical options for depressed scars?**

## DISCUSSION

Anatomically, the lower respiratory tract in the neck consists of the trachea, which is surrounded superiorly by cartilaginous structures of the larynx. The trachea continues inferiorly as a cylindrical tube made up of a flat membrane portion posteriorly and 18 to 22 rigid cartilaginous C-shaped rings anteriorly and laterally. The trachea is protected superficially by strap muscles, subcutaneous tissue, and skin. Surgical tracheostomies are performed between the second and fourth tracheal rings.^1^ All surgical airways are left to heal by secondary intention after decannulation. Granulation tissue forms around the stoma. Epithelium then migrates over the granulation tissue from the wound edges. As the granulation tissue matures, it undergoes fibrosis and contracture, which can depress the final scar. Depression also occurs due to the loss of subcutaneous tissue.^2^

If the contracted scar tissue comes into contact with the trachea, it adheres to the trachea. A depressed and fixed tracheal scar produces “tracheal tug,” which is when the skin and the trachea move concurrently. Tracheal tug can cause dysphagia and pain with either lateral or vertical head movement in addition to an aesthetically unpleasing scar.^2^

To correct hypertrophic tracheostomy scars, the most common procedure is excision of the scar with tensionless closure. Skin closure is performed by simple reapproximation or with local flaps, most commonly a z-plasty. Autologous fat transplantation is a newer, less invasive alternative. Before injecting the fat, fibrotic bands that tether the scar to the underlying tissue are released with a sharp needle.^3^

There are many surgical options to correct depressed tracheal scars associated with tracheal tug. The simplest operation is de-epithelializing the skin over the scar, leaving the underlying scar tissue intact. The surrounding skin is then undermined to ensure tensionless closure. A second option is a subcutaneous z-plasty. The skin and the scar tissue are excised and realigned to decrease tension and raise the level of the scar. However, these 2 techniques do not eliminate tracheal tug. To prevent fibrosis and development of tracheal tug, the skin and the scar tissue can be excised and replaced with a muscle flap, such as the sternocleidomastoid, platysma, or the strap muscles. When using the strap muscles, as in this case, the sternohyoid and sternothyroid muscles are freed from adhesions, approximated, and sutured at the midline. The muscle provides volume to correct scar depression. Maintaining the fascia under the muscle reduces the risk of tracheal adhesions to muscle fibers.^4^ If additional volume is needed, acellular dermal matrix can be placed either above or below the muscle.^5,6^ The scar revision can also be augmented with allogenic dura or autologous fat.^3-8^

Surgical airways can leave debilitating and unsightly scars due to secondary healing and scar formation. Skin closure techniques including scar excision with simple closure or z-plasty are good surgical options to improve aesthetics and neck mobility if limited by contracture. The addition of local muscle flaps or allogenic material is preferred if the scar is associated with tracheal tug.

## Figures and Tables

**Figure 1:**
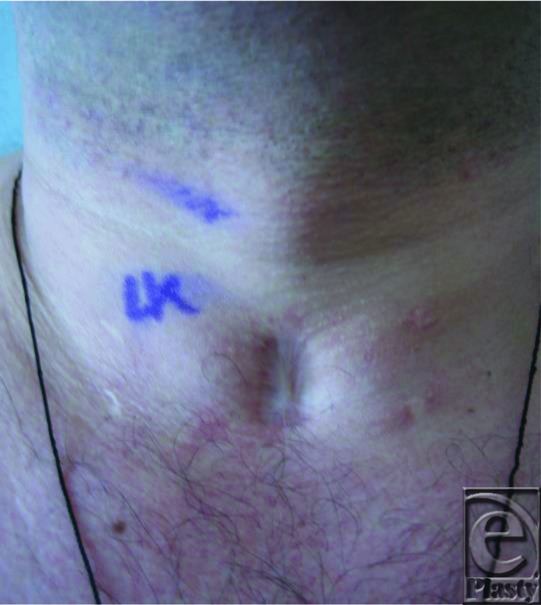
Preoperative photograph of the patient with tracheostomy scar.

**Figure 2:**
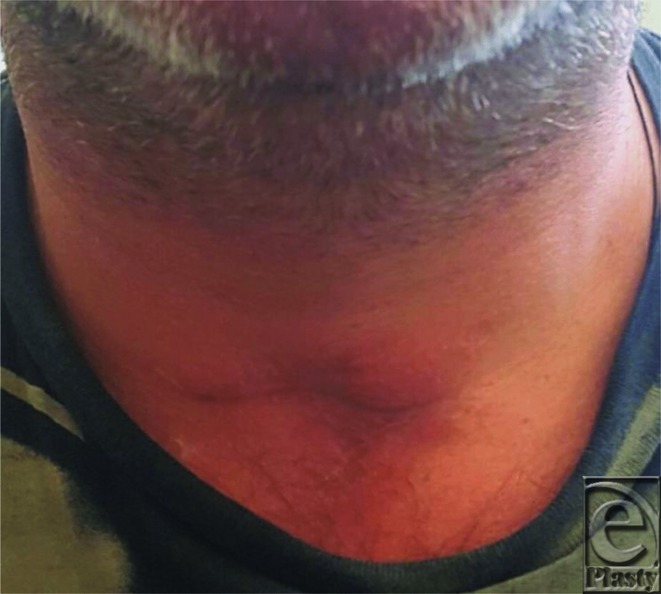
Postoperative photograph after scar revision using strap muscle coverage and z-plasty closure.
